# Clinical factors associated with developmental delay in placental abruption

**DOI:** 10.3389/fmed.2025.1544679

**Published:** 2025-07-02

**Authors:** Eun Hui Joo, Nari Kim, Hyun Mee Ryu, Sang Hee Jung, Eun Hee Ahn, Ji Yeon Lee

**Affiliations:** Department of Obstetrics and Gynecology, CHA Bundang Medical Center, CHA University School of Medicine, Seongnam, Republic of Korea

**Keywords:** placental abruption, placenta, obstetric hemorrhage, developmental delay, postnatal long-term outcomes

## Abstract

**Background:**

This study aimed to investigate the association between clinical characteristics and neonatal developmental delay (DD) in women with placental abruption (PA).

**Methods:**

We retrospectively reviewed obstetric characteristics and perinatal outcomes of singleton pregnancies complicated by PA who were healthy before pregnancy between 2010 and 2021. Neuromotor development was evaluated using Bayley Scales of Infant and Toddler Development, Third Edition, and/or Gross Motor Function Measure. Clinical characteristics were compared between offspring with and without developmental delay to identify associated risk factors.

**Results:**

Among 9,374 deliveries, 188 cases (2.0%) were diagnosed with PA, and 33 infants exhibited developmental delay. Maternal demographics, including age, body mass index (BMI), nulliparity, and history of preterm birth, did not differ significantly between groups. Prenatal ultrasound suspected PA in 16.4% of cases in the developmental delay group and 18.2% in the no-delay group. However, a longer interval between diagnosis and delivery [adjusted OR (aOR) = 9.82; 95% CI, 1.25–77.24; *P* = 0.030] and delivery before 32 weeks' gestation (aOR = 19.65; 95% CI, 1.46–264.40; *P* = 0.025) were significantly associated with developmental delay.

**Conclusion:**

Ultrasound findings suggestive of PA were not associated with developmental delay in offspring. However, a prolonged diagnosis-to-delivery interval and extreme prematurity were significant risk factors. These findings underscore the limitations of ultrasound in detecting clinically significant PA and highlight the importance of timely clinical decision-making. Further research is warranted to improve diagnostic strategies for PA.

## Introduction

Placental abruption (PA) is clinically defined as the premature detachment of a normally implanted placenta from the uterine wall before the delivery of the fetus. This condition can result in bleeding at the decidual-placental interface and poses a serious threat to both maternal and perinatal health, contributing significantly to morbidity and mortality (1–3). Globally, PA occurs in ~1% of all pregnancies (4). Known risk factors include advanced maternal age, parental smoking, a history of previous abruption, high parity, multiple gestations, and obstetric or medical conditions such as polyhydramnios, chorioamnionitis, coagulation disorders, and abdominal trauma (5).

PA can have profound and potentially life-threatening effects on both the mother and the fetus. The clinical severity of PA varies depending on the extent of placental separation, gestational age at onset, and the timeliness of medical intervention (2, 6–8).

Disruption of placental attachment impairs oxygen and nutrient exchange between mother and fetus, increasing the risk of fetal hypoxia. This may lead to complications such as fetal distress, neonatal encephalopathy, or hypoxic-ischemic encephalopathy, which are associated with long-term neurodevelopmental deficits, including cognitive and motor delays (9–12).

Understanding clinical factors that may influence long-term neurodevelopmental outcomes in infants born after PA is essential for improving prognosis and guiding clinical management. However, few studies have systematically evaluated such factors. Therefore, this study aimed to investigate the association between maternal and fetal clinical characteristics and developmental delay (DD) in offspring born from pregnancies complicated by PA.

## Methods

### Patients and study design

In this retrospective cohort study, singleton women with PA who gave birth at CHA Bundang Medical Center from January 2010 to December 2021 were included. In cases with multifetal pregnancies, maternal medical or surgical problems before pregnancy, or congenital fetal anomalies were excluded. PA was diagnosed according to the judgment of the attending physician based on the clinical course, such as abdominal pain with bleeding, abnormal fetal heart pattern on cardiotocography, the existence of retroplacental hematoma on ultrasonography, and other clinical findings suspicious for PA. We divided the cohort into two groups, one with developmental delay (DD group) and the other without developmental delay (No DD group).

### Ethics approval

The institutional review boards (IRB) of CHA Bundang Medical Center approved this study (IRB no.: 2023-11-003, dates of approval: Nov. 17, 2023). Informed consent was waived for this retrospective cohort study as it involved the analysis of medical records. The IRB of the research institute approved the study and determined that obtaining informed consent was not necessary. The study methods strictly adhered to the relevant guidelines and regulations set forth by the IRB at this institution.

### Review of medical records

We reviewed obstetric characteristics such as maternal age, height, body mass index (BMI) before pregnancy, parity, mode of conception, and other medical conditions during pregnancy, perinatal outcomes including meconium aspiration syndrome (MAS), jaundice, transient tachypnea of the newborn (TTN), respiratory distress syndrome (RDS), bronchopulmonary dysplasia (BPD), sepsis, pulmonary hypertension (HTN), intracranial hemorrhage (ICH), neonatal enterocolitis (NEC), retinopathy of prematurity (ROP), and children's neurodevelopmental records.

Small for gestational age (SGA) was evaluated by the guideline of Journal of Korea Medical Science (13), and neuromotor development was evaluated using Bayley-III tests, and/or Gross Motor Function Measure (GMFM). If the test scores indicate a deviation below the normal development reference, it is classified as developmental delay. In cases where a formal developmental screening test was not performed, we relied on information from medical records. This information includes descriptions of the child's developmental status based on brief developmental screening, and age-appropriate questionnaires related to language, thinking, behavior, and movement. Additionally, the child's ability to meet their academic obligations was considered as part of the assessment. If a child reported no difficulties in meeting their academic responsibilities, their development was considered to be within the normal range in this context.

### Statistical analysis

Statistical analysis was conducted using SPSS software (version 28.0; SPSS Institute, Chicago, IL, USA). Categorical variables were analyzed using the Chi-square test, and continuous variables were analyzed using the Student *t*-test. Additionally, univariate and multivariate analyses were performed using explanatory variables to calculate the unadjusted odds ratios (ORs) and adjusted ORs (aORs). Variables associated with neurodevelopmental delay in univariate analyses (*P* < 0.1) and those of clinical relevance were included in the multivariate logistic regression model. Variance inflation factors (VIFs) were calculated to assess multicollinearity among predictor variables. A VIF >5 was considered indicative of collinearity; no significant collinearity was detected. Model fit was evaluated using the Hosmer–Lemeshow goodness-of-fit test. Given the number of comparisons, statistical significance was set at *P* < 0.05 without formal adjustment for multiple comparisons. Sensitivity analyses were performed to assess the robustness of findings, including excluding extreme preterm cases (<28 weeks) and redefining the primary outcome with stricter criteria.

## Results

Among 9,374 singleton deliveries during the study period, 188 cases (2.0%) were diagnosed with placental abruption (PA). Of these, eight resulted in intrauterine fetal demise and one in neonatal death 2 days after birth. Additionally, 55 infants were lost to follow-up, leaving 124 cases available for analysis [attrition rate: 34% (64/188)]. Among these, 33 infants (26.6%) were confirmed to have developmental delay (DD group), while 91 (73.4%) showed no such delay (No DD group).

There were no significant differences in maternal age, pre-pregnancy BMI, parity, history of preterm birth, mode of conception, or pregnancy complications between the DD and No DD groups (Table 1). However, hypertensive disorders of pregnancy were significantly more prevalent in the DD group (24.2 vs. 4.3%, *P* = 0.001).

**Table 1:** Maternal characteristics before and during the pregnancy.

	**No DD group (*n* = 91)**	**DD group (*n* = 33)**	***P-*value**
Age (years)^*^	33.8 ± 3.9	33.5 ± 4.0	0.94
BMI before pregnancy^*^	21.5 ± 3.7	21.3 ± 3.0	0.509
Nulliparity^†^	53 (58.2)	18 (54.5)	0.325
Prior preterm birth^†^	2 (2.2)	1 (3.0)	0.925
**Mode of conception**			
Natural pregnancy^†^	71 (78.0)	26 (78.9)	0.812
Ovarian stimulation^†^	4 (4.4)	1 (3.0)	
*In vitro* fertilization^†^	16 (17.6)	6 (18.2)	
Hypertensive disorders of pregnancy^†^	4 (4.3)	8 (24.2)	0.001
Gestational diabetes mellitus^†^	6 (6.6)	0 (0.0)	0.132
Placenta previa^†^	5 (5.5)	1 (3.0)	0.574
Cerclage operation^†^	4 (4.4)	3 (9.15)	0.319
Antenatal admission due to preterm labor^†^	25 (27.5)	10 (30.3)	0.758

DD, developmental delay.

* Data given as mean ± SD.

† Data given as n (%).

Prenatal suspicion of PA via ultrasound was low in both groups (18.2% in DD vs. 16.4% in No DD, Table 2). The interval from PA diagnosis to delivery was longer in the DD group (203.0 ± 377.4 min) compared to the No DD group (111.5 ± 153.2 min, *P* = 0.041). More DD cases had an interval >150 min (30.3 vs. 7.7%, *P* = 0.023).

**Table 2:** Characteristics of delivery.

	**No DD group (*n* = 91)**	**DD group (*n* = 33)**	***P-*value**
PA diagnosis before delivery by ultrasound^*^	15 (16.4)	6 (18.2)	0.967
Cesarean section^*^	50 (55.4)	29 (87.9)	0.001
Emergency delivery^*^	44 (48.4)	29 (87.9)	<0.001
Estimated blood loss (ml)^†^	693.2 ± 611.3	586.4 ± 291.6	0.389
Interval between diagnosis of PA and delivery (min)^†^	111.5 ± 153.2	203 ± 377.4	0.041
Interval between diagnosis of PA and delivery >150 min^*^	7 (7.7)	10 (30.3)	0.023

PA, placental abruption.

* Data given as n (%).

† Data given as mean ± SD.

Gestational age at delivery was significantly earlier in the DD group (32.8 ± 3.7 weeks) than in the No DD group (37.7 ± 2.2 weeks, *P* < 0.001). Preterm birth rates before 28, 32, 34, and 37 weeks were significantly higher in the DD group (*P* ≤ 0.001, Table 3). Birthweight was significantly lower in the DD group (1,895 ± 759 g vs. 2,889 ± 532 g, *P* = 0.003), although SGA rates did not differ. Neonatal complications including MAS, jaundice, RDS, BPD, pulmonary hypertension, ICH, NEC, and ROP were significantly more frequent in the DD group.

**Table 3:** Obstetric and neonatal outcomes.

	**No DD group (*n* = 91)**	**DD group (*n* = 33)**	***P-*value**
Gestational age at delivery (weeks)^*^	37.7 ± 2.2	32.8 ± 3.7	<0.001
**Preterm birth**			
< 28 weeks^†^	0 (0.0)	4 (12.1)	0.001
< 32 weeks^†^	2 (2.2)	13 (39.4)	<0.001
< 34 weeks^†^	6 (6.6)	17 (51.5)	<0.001
< 37 weeks^†^	28 (30.8)	26 (78.8)	<0.001
Birthweight (g)^*^	2889 ± 532	1895 ± 759	0.003
Small for gestational age^†^	13 (14.3)	6 (18.1)	0.58
Male neonate^†^	46 (50.5)	19 (57.6)	0.457
Apgar score at 1 min^†^	7.3 ± 1.4	5.3 ± 2.2	<0.001
Apgar score at 5 min^†^	8.5 ± 1.2	6.8 ± 2.2	<0.001
Apgar score at 5 min < 7^†^	6 (5.5)	13 (39.4)	<0.001
NICU admission^†^	30 (33.0)	29 (87.9)	<0.001
NICU hospitalization (day)^*^	17.6 ± 15.4	52.9 ± 47.0	<0.001
MAS^†^	7 (7.7)	8 (24.2)	0.013
Jaundice^†^	42 (46.2)	24 (72.7)	0.009
TTN^†^	14 (15.4)	6 (18.2)	0.709
RDS^†^	5 (5.5)	15 (45.5)	<0.001
BPD^†^	0 (0.0)	7 (21.2)	<0.001
Pulmonary hypertension^†^	0 (0.0)	3 (9.1)	0.004
ICH^†^	4 (4.4)	10 (30.3)	<0.001
NEC^†^	0 (0.0)	4 (12.1)	0.001
Sepsis^†^	12 (13.2)	4 (12.1)	0.876
ROP^†^	0 (0.0)	5 (15.2)	<0.001

NICU, neonatal intensive care unit; MAS, meconium aspiration; TTN, transient tachypnea of neonate; RDS, respiratory distress syndrome; BPD, bronchopulmonary dysplasia; ICH, Intracranial hemorrhage; NEC, Necrotizing enterocolitis; ROP, retinopathy of prematurity.

* Data given as mean ± SD.

† Data given as n (%).

Multivariate analysis showed that a time interval >150 min from PA diagnosis to delivery (aOR = 9.82; 95% CI, 1.25–77.24; *P* = 0.030) and preterm birth before 32 weeks of gestation (aOR = 19.65; 95% CI, 1.46–264.40; *P* = 0.025) were independently associated with developmental delay (Table 4). Sensitivity analyses excluding cases <28 weeks GA and redefining developmental delay to include only standardized assessments did not substantially alter the direction or significance of the primary associations.

**Table 4:** Multiple regression analysis of obstetric factors that affected developmental delay.

	**Unadjusted odds ratio (95% CI)**	***P*-value**	**Adjusted odds ratio (95% CI)**	***P*-value**
Interval between diagnosis of PA and delivery >150 min	3.67 (1.17–11.56)	0.026	9.82 (1.25–77.24)^a^	0.030
Preterm birth < 32 weeks	29.25 (6.11–139.97)	<0.0001	19.65 (1.46–264.40)^b^	0.025
Preterm birth < 34 weeks	15.23 (5.21–44.53)	<0.0001	10.12 (0.95–108.12)^b^	0.056
Preterm birth < 37 weeks	8.49 (3.30–21.85)	<0.0001	3.14 (0.41–24.23)^b^	0.273

PA, placental abruption; CI, confidence interval.

a Adjusted for maternal age, BMI before pregnancy, parity, previous preterm birth, mode of conception, gestational hypertension, gestational age at delivery, and emergency cesarean section.

b Adjusted for maternal age, BMI before pregnancy, parity, previous preterm birth, mode of conception, gestational hypertension, interval between diagnosis of PA and delivery >150 min, and emergency cesarean section.

## Discussion

### Principal findings

This study reveals specific clinical factors associated with developmental delay in neonates born from pregnancies complicated by PA, a relationship that has not yet been well-explored in previous studies. We found that a diagnosis-to-delivery interval exceeding 150 min and delivery before 32 weeks were significant risk factors for developmental delay. These findings highlight the importance of timely recognition and intervention in PA cases to improve long-term neurodevelopmental outcomes.

### Review in the context of what is known

Previous studies have primarily focused on the clinical presentation and risk factors of PA, emphasizing its association with adverse neonatal outcomes such as low Apgar scores, intrauterine fetal death, RDS, and neonatal intensive care unit admission (14–16). Furthermore, several investigations have linked PA to long-term neurodevelopmental complications in offspring, including cerebral palsy and developmental delay. For example, Pariente et al. and Oltean et al. (9, 10) reported significantly higher odds of cerebral palsy in infants born to women with PA compared to those without. However, most of these studies did not address the impact of the time interval between PA diagnosis and delivery on neurodevelopmental outcomes. This gap is clinically relevant, as a prolonged interval may exacerbate fetal hypoxia due to impaired placental perfusion, which is a key mechanism in hypoxic-ischemic brain injury and subsequent developmental delay. In our study, we observed that a longer diagnosis-to-delivery interval was significantly associated with developmental delay, independent of gestational age and other risk factors. These findings underscore the importance of prompt obstetric intervention following suspected PA to minimize neonatal neurodevelopmental sequelae.

Our findings also reinforce the well-documented limitations of ultrasonography in diagnosing PA. The rate of antepartum diagnosis using ultrasound did not differ significantly between the DD and non-DD groups (18.2 vs. 16.4%, *P* = 0.967), with the majority of cases diagnosed clinically based on symptoms such as vaginal bleeding, abdominal pain, and fetal heart rate abnormalities. Conventional ultrasound is known to have high positive predictive value (PPV: 88%−100%) but low sensitivity (23%−57%) in detecting PA (17, 18). This limitation is due in part to the isoechogenic nature of acute hematomas and the variability in hematoma size, location, and timing relative to the abruption event. Because ultrasound has limited sensitivity for detecting small or acute retroplacental hematomas, negative findings cannot reliably exclude PA (19). Recent studies have attempted to overcome these diagnostic challenges by incorporating adjunctive imaging modalities. For instance, Shih et al. (20) demonstrated that the addition of color Doppler significantly improved the sensitivity of ultrasound in detecting PA. Similarly, Agrawal et al. (21) emphasized the utility of serial imaging combined with maternal serum markers to enhance diagnostic accuracy. These findings point to a growing consensus favoring multi-modal diagnostic strategies rather than sole reliance on conventional ultrasound.

Despite the increasing attention to neonatal outcomes following PA, the literature on long-term neurodevelopment remains limited and inconsistent. Discrepancies across studies may be attributed to heterogeneous definitions of developmental delay, variable follow-up durations, and differing assessment tools. While some reports, such as Ananth et al. (22), suggest a heightened risk of cerebral palsy and cognitive impairment, others found no significant cognitive deficits after controlling for gestational age and birth weight at follow-up beyond age five (9). These inconsistencies underscore the need for large-scale, prospective cohort studies using standardized outcome definitions. Current guidelines advocate for structured, standardized neurodevelopmental follow-up protocols in high-risk neonates, such as those exposed to PA, to ensure early detection and intervention (23, 24). Future research should aim to harmonize follow-up practices and assessment methods to allow more robust comparisons and evidence-based recommendations.

### Clinical applications

This study contributes valuable clinical insights into the correlation between the prenatal diagnosis of PA and adverse pregnancy outcomes, specifically developmental delay in newborns. The limited occurrence of suspected PA cases identified by ultrasound before delivery indicates that depending solely on ultrasound may not be adequate for early PA detection. Consequently, clinicians should explore additional clinical indicators and risk factors associated with PA to improve early identification and ensure appropriate management. Moreover, the study suggests the need for further research to establish new indicators and predictive models related to PA, aligning with advancements in ultrasound technology.

### Strengths and limitations

This study has several strengths. Most notably, it is the first, to our knowledge, to investigate clinical factors associated with developmental delay in offspring following pregnancies complicated by PA. The analysis was conducted within a single tertiary center over a 12-year period, ensuring consistency in clinical management and data collection. Additionally, key findings–particularly the associations between prolonged diagnosis-to-delivery interval, extreme prematurity, and neurodevelopmental outcomes–were supported by sensitivity analyses, underscoring the robustness of the observed relationships.

However, the study also has important limitations. First, its retrospective design introduces inherent risks of bias and confounding, including the inability to fully adjust for unmeasured variables such as socioeconomic status, postnatal care quality, and maternal health. Second, the definition of developmental delay was heterogeneous, incorporating both standardized tools (e.g., Bayley-III, GMFM), and informal assessments (e.g., clinician judgment, educational records), which may reduce the validity and reliability of outcome measures. Third, the high attrition rate (34%) due to long-term loss to follow-up may have introduced selection bias and limits the generalizability of the findings, as children lost to follow-up could differ systematically from those retained in the cohort.

From a statistical perspective, the study was limited by a relatively small sample size, resulting in wide confidence intervals for some estimates and limiting the precision of the findings. While multicollinearity was ruled out using variance inflation factors, the lack of multiple comparison correction may increase the risk of type I error. Furthermore, the absence of a pre-specified power calculation reduces the strength of inferences drawn from non-significant results. Future prospective studies with larger sample sizes and standardized outcome assessments are needed to validate these findings and better elucidate the mechanisms linking PA to adverse neurodevelopmental outcomes.

## Conclusion

In this cohort of infants born following PA, a prolonged interval between diagnosis and delivery, as well as extreme prematurity, were significantly associated with an increased risk of neurodevelopmental delay. In contrast, ultrasound findings suggestive of PA were not predictive of developmental outcomes, underscoring the limitations of current imaging modalities in guiding perinatal decision-making. These findings highlight the importance of timely clinical assessment and decision-making in cases of suspected PA. Enhanced prenatal surveillance and rapid intervention strategies may be essential to reduce the risk of long-term neurodevelopmental impairment. Future prospective studies are warranted to refine diagnostic tools and optimize perinatal management.

## Data Availability

The raw data supporting the conclusions of this article will be made available by the authors, without undue reservation.

## References

[1] Pariente G Wiznitzer A Sergienko R Mazor M Holcberg G Sheiner E. Placental abruption: critical analysis of risk factors and perinatal outcomes. J Matern Fetal Neonatal Med. (2011) 24:698–702. 10.3109/14767058.2010.511346 20828239

[2] Ananth CV Lavery JA Vintzileos AM Skupski DW Varner M Saade G. Severe placental abruption: clinical definition and associations with maternal complications. Am J Obstet Gynecol. (2016) 214:272.e1-.e9. 10.1016/j.ajog.2015.09.069 26393335

[3] Downes KL Grantz KL Shenassa ED. Maternal, labor, delivery, and perinatal outcomes associated with placental abruption: a systematic review. Am J Perinatol. (2017) 34:935–57. 10.1055/s-0037-1599149 28329897 PMC5683164

[4] Hossain N Khan N Sultana SS Khan N. Abruptio placenta and adverse pregnancy outcome. J Pak Med Assoc. (2010) 60:443–6. 20527640

[5] Minire A Mirton M Imri V Lauren M Aferdita M. Maternal complications of preeclampsia. Med Arch. (2013) 67:339–41. 10.5455/medarh.2013.67.339-341 24601166

[6] Räisänen S Gissler M Saari J Kramer M Heinonen S. Contribution of risk factors to extremely, very and moderately preterm births - register-based analysis of 1,390,742 singleton births. PLoS ONE. (2013) 8:e60660. 10.1371/journal.pone.0060660 23577142 PMC3618176

[7] Riihimäki O Metsäranta M Ritvanen A Gissler M Luukkaala T Paavonen J. Increased prevalence of major congenital anomalies in births with placental abruption. Obstet Gynecol. (2013) 122:268–74. 10.1097/AOG.0b013e31829a6f91 23969794

[8] Kayani SI Walkinshaw SA Preston C. Pregnancy outcome in severe placental abruption. BJOG. (2003) 110:679–83. 10.1046/j.1471-0528.2003.02088.x 12842059

[9] Oltean I Rajaram A Tang K MacPherson J Hondonga T Rishi A. The association of placental abruption and pediatric neurological outcome: a systematic review and meta-analysis. J Clin Med. (2022) 12:205. 10.3390/jcm12010205 36615006 PMC9821447

[10] Pariente G Wainstock T Walfisch A Landau D Sheiner E. Placental abruption and long-term neurological hospitalisations in the offspring. Paediatr Perinat Epidemiol. (2019) 33:215–22. 10.1111/ppe.12553 31131918

[11] Nasiell J Papadogiannakis N Löf E Elofsson F Hallberg B. Hypoxic ischemic encephalopathy in newborns linked to placental and umbilical cord abnormalities. J Matern Fetal Neonatal Med. (2016) 29:721–6. 10.3109/14767058.2015.1015984 25714479

[12] Locatelli A Incerti M Paterlini G Doria V Consonni S Provero C. Antepartum and intrapartum risk factors for neonatal encephalopathy at term. Am J Perinatol. (2010) 27:649–54. 10.1055/s-0030-1249761 20225171

[13] Lee JK Jang HL Kang BH Lee KS Choi YS Shim KS. Percentile distributions of birth weight according to gestational ages in Korea (2010-2012). J Korean Med Sci. (2016) 31:939–49. 10.3346/jkms.2016.31.6.939 27247504 PMC4853674

[14] Jeong YY Yun TH Hong SY Bae JY. Significance of clinical findings of patients with placental abruption. Perinatology. (2020) 31:123–8. 10.14734/PN.2020.31.3.123

[15] Downes KL Shenassa ED Grantz KL. Neonatal outcomes associated with placental abruption. Am J Epidemiol. (2017) 186:1319–28. 10.1093/aje/kwx202 28595292 PMC5860509

[16] Eubanks AA Walz S Thiel LM. Maternal risk factors and neonatal outcomes in placental abruption among patients with equal access to health care. J Mater Fetal Neonat Med. (2021) 34:2101–6. 10.1080/14767058.2019.1657088 31416373

[17] Glantz C Purnell L. Clinical utility of sonography in the diagnosis and treatment of placental abruption. J Ultrasound Med. (2002) 21:837–40. 10.7863/jum.2002.21.8.837 12164566

[18] Shinde GR Vaswani BP Patange RP Laddad MM Bhosale RB. Diagnostic performance of ultrasonography for detection of abruption and its clinical correlation and maternal and foetal outcome. J Clin Diagn Res. (2016) 10:Qc04–7. 10.7860/JCDR/2016/19247.8288 27656507 PMC5028521

[19] Kaakaji Y Nghiem HV Nodell C Winter TC. Sonography of obstetric and gynecologic emergencies: Part II, Gynecologic emergencies. AJR Am J Roentgenol. (2000) 174:651–6. 10.2214/ajr.174.3.1740651 10701603

[20] Shih J Jaraquemada JP Su Y Shyu M Lin C Lin S. Role of three-dimensional power Doppler in the antenatal diagnosis of placenta accreta: comparison with gray-scale and color Doppler techniques. Ultra Obstet Gynecol. (2009) 33:193–203. 10.1002/uog.6284 19173239

[21] Agrawal S Parks WT Zeng HD Ravichandran A Ashwal E Windrim RC. Diagnostic utility of serial circulating placental growth factor levels and uterine artery Doppler waveforms in diagnosing underlying placental diseases in pregnancies at high risk of placental dysfunction. Am J Obstet Gynecol. (2022) 227:618. e1-. e16. 10.1016/j.ajog.2022.05.043 35644246

[22] Ananth CV Hansen AV Elkind MS Williams MA Rich-Edwards JW Nybo Andersen A-M. Cerebrovascular disease after placental abruption: a population-based prospective cohort study. Neurology. (2019) 93:e1148–e58. 10.1212/WNL.0000000000008122 31420459

[23] Merchant N Shekhar L Huertas-Ceballos A Johnson S Arasu A. Neurodevelopmental care following neonatal discharge. Paediatr Child Health. (2022) 32:324–31. 10.1016/j.paed.2022.07.002

[24] Malak R Kaczmarek A Fechner B Samborski W Kwiatkowski J Komisarek O. The importance of follow-up visits for children at risk of developmental delay—a review. Diagnostics. (2024) 14:1764. 10.3390/diagnostics14161764 39202251 PMC11354016

